# Case fatality rate among COVID-19 patients treated with acute kidney replacement therapy

**DOI:** 10.1590/2175-8239-JBN-2022-0161en

**Published:** 2023-11-13

**Authors:** Gabriel Martins Nogueira, Paulo Novis Rocha, Constança Margarida Sampaio Cruz

**Affiliations:** 1 Escola Bahiana de Medicina e Saúde Pública, Departamento de Medicina, Salvador, BA, Brazil.; 2 Universidade Federal da Bahia, Faculdade de Medicina da Bahia, Salvador, BA, Brazil.

**Keywords:** Acute Kidney Injury, Kidney Replacement Therapy, COVID-19, Injúria Renal Aguda, Suporte Renal Artificial, COVID-19

## Abstract

**Introduction:**

Acute kidney injury (AKI) is a frequent complication of severe COVID-19 and is associated with high case fatality rate (CFR). However, there is scarcity of data referring to the CFR of AKI patients that underwent kidney replacement therapy (KRT) in Brazil. The main objective of this study was to describe the CFR of critically ill COVID-19 patients treated with acute kidney replacement therapy (AKRT).

**Methods:**

Retrospective descriptive cohort study. We included all patients treated with AKRT at an intensive care unit in a single tertiary hospital over a 15-month period. We excluded patients under the age of 18 years, patients with chronic kidney disease on maintenance dialysis, and cases in which AKI preceded COVID-19 infection.

**Results:**

A total of 100 out of 1479 (6.7%) hospitalized COVID-19 patients were enrolled in this study. The median age was 74.5 years (IQR 64 – 82) and 59% were male. Hypertension (76%) and diabetes mellitus (56%) were common. At the first KRT prescription, 85% of the patients were on invasive mechanical ventilation and 71% were using vasoactive drugs. Continuous veno-venous hemodiafiltration (CVVHDF) was the preferred KRT modality (82%). CFR was 93% and 81 out of 93 deaths (87%) occurred within the first 10 days of KRT onset.

**Conclusion:**

AKRT in hospitalized COVID-19 patients resulted in a CFR of 93%. Patients treated with AKRT were typically older, critically ill, and most died within 10 days of diagnosis. Better strategies to address this issue are urgently needed.

## Resumo

**Introdução:**

Injúria renal aguda (IRA) é uma complicação frequente da COVID-19 grave e está associada a alta taxa de letalidade (TL). Entretanto, há escassez de dados referentes à TL de pacientes com IRA submetidos a suporte renal artificial (SRA) no Brasil. O objetivo principal deste estudo foi descrever a TL de pacientes graves com IRA por COVID-19 tratados com SRA.

**Métodos:**

Estudo de coorte descritivo retrospectivo. Incluímos todos os pacientes tratados com SRA em unidade de terapia intensiva de um único hospital terciário por 15 meses. Excluímos pacientes menores de 18 anos, pacientes com doença renal crônica em diálise de manutenção e casos nos quais a IRA precedeu a infeção por COVID-19.

**Resultados:**

Incluímos neste estudo um total de 100 dos 1479 (6,7%) pacientes hospitalizados com COVID-19. A mediana de idade foi 74,5 anos (IIQ 64 – 82) e 59% eram homens. Hipertensão (76%) e diabetes mellitus (56%) foram comuns. Na primeira prescrição de SRA, 85% dos pacientes estavam em ventilação mecânica invasiva e 71% em uso de drogas vasoativas. A hemodiafiltração contínua foi a modalidade de SRA preferida (82%). A TL foi de 93% e 81 dos 93 óbitos (87%) ocorreram nos primeiros 10 dias do início da SRA.

**Conclusão:**

O SRA em pacientes hospi­talizados com IRA por COVID-19 resultou em TL de 93%. Os pacientes tratados com SRA eram geralmente idosos, gravemente enfermos e a maioria foi a óbito em até 10 dias após o diagnóstico. Estratégias melhores para abordar esse problema são urgentemente necessárias.

## Introduction

COVID-19 is a coronavirus disease caused by the SARS-CoV-2 virus, whose dissemination began in the Chinese province of Wuhan in late 2019^1^. The renal system is seriously impacted by this illness, especially in symptomatic cases, as reflected by the fact that 25% of these patients present with abnormal serum creatinine at hospital admission^2^.

The most severe form of kidney involvement in COVID-19 is acute kidney injury (AKI), defined according to the 2012 KDIGO guidelines as at least one of the following: a) a rise of 0.3 mg/dL in serum creatinine in 48 hours; b) a rise of 1.5 times the basal level of serum creatinine in 7 days; or c) urine output lower than 0.5 mL/kg/h for 6 hours^3^. The pathophysiology of AKI in COVID-19 is most likely multifactorial and involves direct kidney infection by SARS-CoV-2 with subsequent tubular injury^4,5,6,7,8,9,10^, circulatory shock and release of nephrotoxins in the blood^11,12^, overstimulation of the renin-angiotensin-aldosterone system (RAAS)^13,14,15,16,17,18,19^, organ crosstalk between the kidneys, heart, and lungs^20,21,22^, and cytokine release syndrome^23^, among other mechanisms^11^.

The incidence of AKI in COVID-19 varies significantly across studies, ranging from 4 to 75%^24,25,26,27,28,29,30^. However, the largest study we found estimated that approximately one-third of hospitalized patients develop this condition^24^. Of these, around 14% require kidney replacement therapy (KRT). Therefore, the incidence of patients treated with acute kidney replacement therapy (AKRT) is around 5% of all hospitalized COVID-19 patients^24^. In patients admitted only to an intensive care unit (ICU), AKRT is used in 20% of the patients^25^. Risk factors for use of AKRT included male sex, hypertension, diabetes mellitus, chronic kidney disease (CKD), elevated body mass index, high levels of D-dimer and greater severity of hypoxia at admission^25^.

In general, around two in every three patients with COVID-19 that are treated with AKRT expire^25,31^. Additionally, AKRT is considered a risk factor for death in ICU patients^30^. Case fatality rate (CFR) falls to approximately 35% when AKI is managed conservatively, considering both KRT and non-KRT cases^24^. Predictors of 28-day mortality in AKRT include advanced age and severe oliguria^25^. We were able to find two Brazilian studies addressing AKI mortality in COVID-19. The study by Zamoner et al.^30^, set in the state of São Paulo (southeastern region of Brazil), found a CFR of 88% in AKRT patients. The research conducted by Samaan et al.^28^, also in São Paulo (southeastern region of Brazil), evaluated AKRT mortality in COVID-19 and reported that 72.5% of the patients expired. Therefore, there is a significant gap in the Brazilian literature regarding mortality in COVID-19-associated AKRT, especially in patients from the northeastern region of Brazil.

The main objective of this study was to determine the CFR of AKRT in COVID-19 patients admitted to an ICU. Our secondary goals were to explore independent predictors of mortality, identify the median survival time of AKRT patients, and calculate the incidence of AKRT in a hospital setting.

## Methods

### Design and Sample

This was an analytical retrospective cohort study. We included all patients with COVID-19 and AKRT admitted to the intensive care unit of a single tertiary hospital in the city of Salvador, Bahia, Brazil, between the 1^st^ of April 2020 and the 20^th^ of July 2021, including immunosuppressed and transplanted patients. Pregnant women were not included in this study. A COVID-19 diagnosis required a positive reverse transcription polymerase chain reaction. We excluded patients under the age of 18 years, patients with stage 5 CKD on maintenance dialysis, and cases in which AKI preceded COVID-19 infection.

### Data Collection

Initially, the Information and Technology (IT) Department of the hospital released an Excel sheet with all patients diagnosed with COVID-19 that were admitted to the institution between the 1^st^ of April, 2020, and the 20^th^ of July, 2021; the sheet also contained information on whether or not the patient was prescribed AKRT during the hospitalization. Thereafter, we performed a thorough review of electronic medical records of the patients who were prescribed AKRT and applied exclusion criteria. For eligible patients, data were extracted from electronic medical records during August and September 2021.

### Statistical Analysis

Independent categorical variables were described in absolute and relative frequencies, while numeric ones were classified as normal or skewed accor­ding to the Kolmogorov-Smirnoff test. Normally distributed numeric variables were described using mean and standard deviation (SD), while skewed numeric variables were summarized using median and interquartile range (IQR). The Kaplan-Meier estimator was used to report the survival function of the sample.

For the analysis of prognostic markers, patients were divided into two groups according to their life status at discharge (alive or expired). Univariate analysis for the association between death and independent variables involved the use of Fisher’s exact test, Student’s t-test, and Mann-Whitney U-test. Multivariate analysis was not possible due to a large discrepancy between the size of the expired alive groups. Variables with 5% or more of missing data were excluded from the analysis of risk factors for death. All statistical tests were performed on the Statistical Package for the Social Sciences (SPSS), version 14.0.

### Ethical Issues

This study was in accordance with the Declaration of Helsinki and with the resolution 466 of the National Ethics Committee for Research of Brazil (CONEP) and was approved by the Institutional Review Board of the Bahiana School of Medicine and Public Health on the 19^th^ of May 2021.

## Results

**Figure 1.**
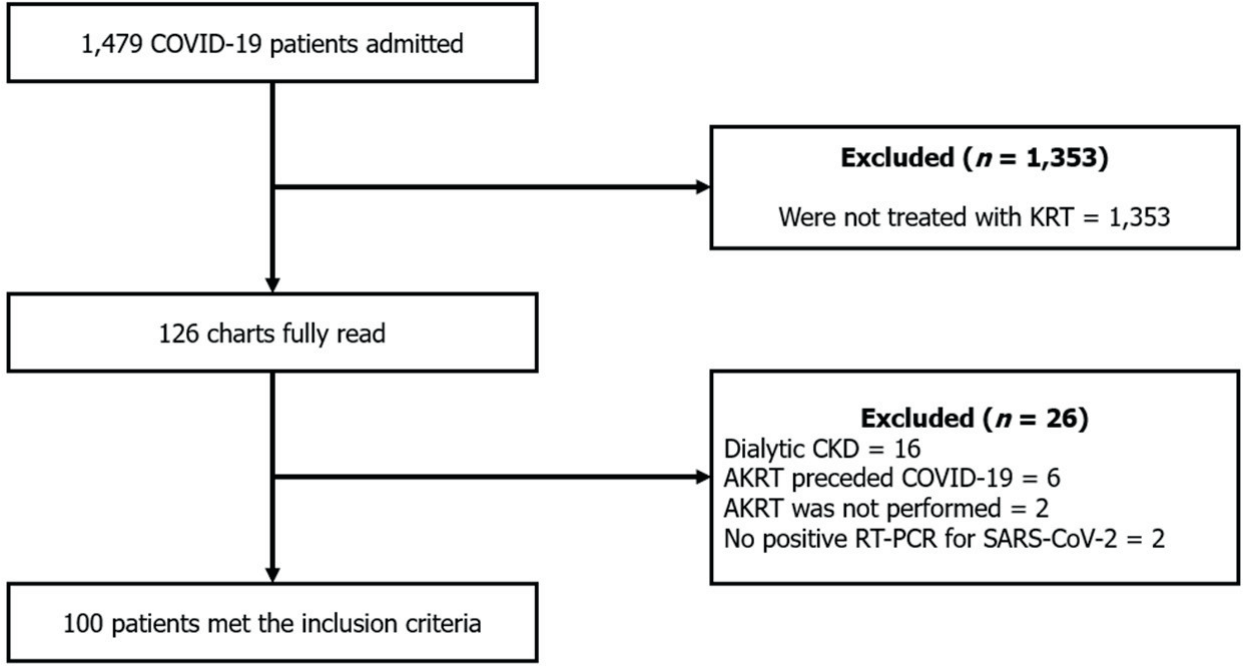
Application of the inclusion and exclusion criteria. **KRT**: Kidney replacement therapy. **CKD**: chronic kidney disease. **AKRT**: acute kidney replacement therapy. **RT-PCR**: reverse transcription polymerase chain reaction.

A total of 1,479 patients were admitted to the institution between the 1^st^ of April, 2020, and the 20^th^ of July, 2021, due to COVID-19. Among these, 126 had at least one AKRT prescription. After applying exclusion criteria, 26 individuals were excluded, leaving 100 (6.7%) AKRT patients for analysis (Figure 1).

Most patients were male (59%) and had a median age of 74.5 years (IQR 64 – 82). Among the comorbidities surveyed, systemic arterial hypertension (76%) and diabetes mellitus (44%) were the most common (Table 1). About 85% of the patients were under invasive mechanical ventilation (IMV) at the time of first AKRT prescription, and remained on this treatment for a median of 11 days (IQR 8 – 15). Moreover, 71% of patients were under the use of at least one vasoactive drug such as norepinephrine or dobutamine. The most used KRT modality was continuous veno-venous hemodiafiltration (CVVHDF), followed by sustained low-efficiency dialysis (SLED), and conventional hemodialysis. Only 6% of all patients discontinued KRT before either death or hospital discharge (Table 2).

**Table 1:** Sample Characteristics

Sociodemographic and Comorbidities	
Male sex	59 [59%]
Age, years (median [IQR])	74.5 [64 – 82]
Systemic arterial hypertension	76 [76%]
Diabetes mellitus	44 [44%]
Dyslipidemia	23 [23%]
Neoplasia	12 [12%]
Arrythmia	10 [10%]
Myocardial infarction, coronary disease, or heart failure	19 [19%]
Stroke	9 [9%]
Chronic respiratory illness	9 [9%]
Hepatobiliary disease	3 [3%]
Rheumatologic disease	4 [4%]
Non-dialysis CKD	13 [13%]
Obesity	16 [16%]
Smoking	15 [15%]

**Table 2:** Variables Related to Hospital Stay

Hospital Stay	
Use of vasoactive drugs	71 (71%)
Time spent in the ICU, days (median [IQR])	16 [10.2 – 24.8]
AKRT duration, days (median [IQR])	4 [2 – 8]
Use of IMV	85 [85%]
Time spent in IMV (median [IQR])	11 [8 – 15.5]
12-hour urine output (median [IQR])	300 [100 – 650]*
Conventional hemodialysis patients	11 [11%]
SLED patients	42 [42%]
CVVHDF patients	82 [82%]
KRT discharge	6 [6%]

Laboratory studies before the first KRT session are shown in Table 3. Complete blood count alterations included decreased values of hemoglobin (mean of 10.2 mg/dL, SD ± 2.2), hematocrit (mean of 30.2%, SD ± 6.7) and leukocytosis (median of 18,660/mm^3^, IQR 13,700 – 22,980) due to neutrophilia (16,453/mm^3^, IQR 11,975 – 20,166).

**Table 3:** Laboratory Exams Before 1^st^ KRT Session

Laboratory Exams Before 1^ST^ KRT Session	
Hemoglobin, mg/dL (mean + SD)	10.2 + 2.2
Hematocrit, % (mean + SD)	30.2 + 6.7
Leucocyte count, 10^3^/mm^3^ (median [IQR])	18.66 [13.70 – 22.98]
Banded neutrophils, 10^3^/mm^3^ (median [IQR])	0 [0 – 606.6]
Segmented neutrophils, 10^3^/mm^3^ (median [IQR])	16.5 [12.0 – 20.2]
Lymphocytes, 10^3^/mm^3^ (median [IQR])	858.9 [532.2 – 1511.2]
Serum urea, mg/dL (mean ± SD)	240.1 + 78.9
Serum creatinine, mg/dL (median [IQR])	3.3 [2.2 – 4.7]
Urea-creatinine ratio (median [IQR])	72.1 [44.7 – 109.4]
Serum sodium, mmol/L (mean ± SD)	134.0 + 8.7
Serum potassium, mmol/L (median [IQR])	4.6 [4.0 – 5.3]
Ionized calcium, mmol/L (median [IQR])	1.05 [1.00 – 1.15]
Serum phosphorus, mg/dL (median [IQR])	5.8 [4.3 – 7.7]
Serum magnesium, mg/dL (median [IQR])	2.3 [2.0 – 2.6]
Serum bicarbonate, mmol/L (median [IQR])	20.8 [18.5 – 24.9]
Serum lactate, mmol/L (median [IQR])	2.4 [1.7 – 3.1]
Proteinuria	62 [87%]**
Hematuria	54 [76%]**
Urinary casts	13 [18%]**
D-dimer, mcg/dL (median [IQR])	3.3 [1.7 – 7.2]
ALT, U/L (median [IQR])	31 [23 – 65,20]
AST, U/L (median [IQR])	54 [38 – 82,50]
C-reactive protein, mg/dL (median [IQR])	6.2 [3.8 – 18.5]
Troponin I, ng/mL (median [IQR])	0.15 [0.06 – 0.52]

**ALT**: alanine transaminase. **AST**: aspartate transaminase.

* Based on 76/100 records; **Based on 71/100 records.

Kidney dysfunction is manifested through increased values of serum urea (mean of 240.1 mg/dL, SD ± 78.9) and serum creatinine (median of 3.3 mg/dL, IQR 2.2 – 4.7). The urea-creatinine ratio was also increased (median of 72.1, IQR 44.7 – 109.4). Regarding electrolytes, we documented low levels of serum ionized calcium (median of 1.05 mmol/L, IQR 1 – 1.1) and high levels of serum phosphorus (median of 5.8 mg/dL, IQR 4.3 – 7.7). We also found decreased values of serum bicarbonate (median of 20.8 mmol/L, IQR 18.5 – 24.9) and increased values of serum lactate (median of 2.4 mmol/L, IQR 1.7 – 3.1). Other alterations were elevated levels of D-dimer (median of 3.3 mcg/dL, IQR 1.7 – 7.2), C-reactive protein (median of 6.2 mg/dL, IQR of 3.8 – 18.5), and troponin I (median of 0.15 ng/mL, IQR 0.06 – 0.52).

**Figure 2.**
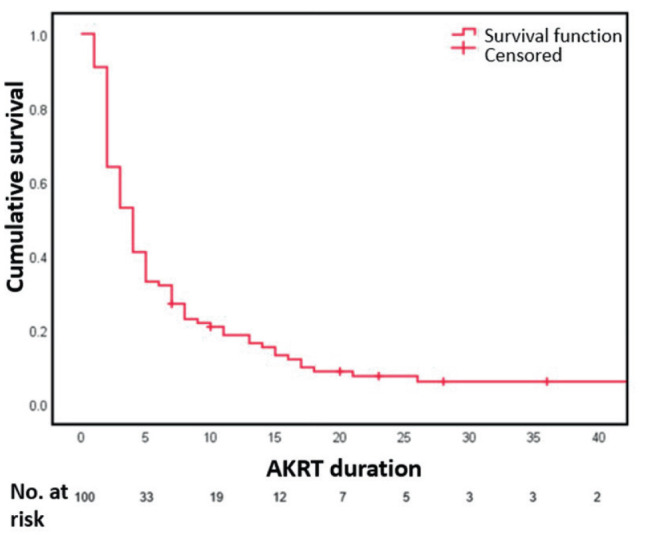
Kaplan-Meier survival function. **AKRT**: acute kidney replacement therapy.

The CFR was 93%. When we analyzed the survival function of our sample, we found that nearly all deaths occur in the first twenty days of AKRT onset. The first ten days of AKRT onset were decisive for either expiration or discharge for 85% of our patients. This results in a short median time of AKI duration (4 days, IQR 2 – 8). The cumulative survival is shown in Figure 2.

Univariate analysis showed an association between death and the following variables: time spent in the ICU (p = 0.005), AKI duration (p = 0.000), use of conventional hemodialysis (p = 0.028), use of SLED (p = 0.002), KRT discharge (p = 0.000), ionized calcium (p = 0.042), C-reactive protein (p = 0.05), and troponin I (p = 0.043). As previously established, these variables were included in a multivariate logistic regression model for analysis of independent association with death (p < 0.05, but no independent association was found. The full univariate analysis is shown in Table 4.

**Table 4:** Univariate Analysis

Variable	Non-expired (n = 7)	Expired (n = 93)	p-value
Male sex	5 (71%)	54 (58%)	0.697
Age, years (median [IQR])	61 (51 – 82)	75 (65 – 82)	0.242
Systemic arterial hypertension	4 (57%)	72 (77%)	0.354
Diabetes mellitus	3 (43%)	41 (44%)	1.000
Dyslipidemia	1 (14%)	22 (24%)	1.000
Neoplasia	1 (14%)	11 (12%)	1.000
Arrythmia	0 (0%)	10 (11%)	1.000
Myocardial infarction, coronary disease, or heart failure	2 (29%)	17 (18%)	0.615
Stroke	0 (0%)	9 (10%)	1.000
Chronic respiratory illness	0 (0%)	9 (10%)	1.000
Hepatobiliary disease	1 (14%)	2 (2%)	0.197
Rheumatologic disease	0 (0%)	4 (4%)	1.000
Non-dialysis CKD	1 (14%)	12 (13%)	1.000
Obesity	1 (14%)	15 (16%)	1.000
Smoking	0 (0%)	15 (16%)	0.590
Use of vasoactive drugs	4 (57%)	67 (72%)	0.410
Time spent in the ICU, days (median [IQR])	43 (14 – 70)	16 (10 – 23.5)	0.005
AKI duration, days (median [IQR])	23 (10 – 36)	3 (2 – 7)	0.000
Use of IMV	6 (86%)	79 (85%)	1.000
Time spent in IMV (median [IQR])	11 (9.75 – 65.50)	11 (8 – 15)	0.232
Conventional hemodialysis patients	3 (43%)	8 (9%)	0.028
SLED patients	7 (100%)	35 (38%)	0.002
CVVHDF patients	4 (57%)	78 (84%)	0.108
Change from conventional hemodialysis or SLED to CVVHDF	2 (29%)	21 (23%)	0.659
Change from CVVHDF to conventional hemodialysis or SLED	3 (43%)	8 (9%)	0.028
KRT discharge	5 (71%)	1 (1%)	0.000
Hemoglobin (mean ± SD)	9.8 ± 2.7	10.2 ± 2.2	0.642
Hematocrit (mean ± SD)	28.76 ± 7.6	30.36 ± 6.6	0.543
Leucocyte count, 10^3^/mm^3^ (median [IQR])	19.1 (11.5 – 20.3)	18.5 (13.7 – 23.1)	0.487
Lymphocytes, 10^3^/mm^3^ (median [IQR])	0.7 (0.3 – 1.3)	0.9 (0.5 – 1.6)	0.487
Segmented neutrophils, 10^3^/mm^3^ (median [IQR])	17.0 (10.3 – 18.1)	16.3 (12.2 – 20.3)	0.730
Bands, 10^3^/mm^3^ (median [IQR])	0 (0 – 1.2)	0 (0 – 0.6)	0.753
Serum urea, mg/dL (mean ± SD)	246.0 (± 69.3)	239.6 ± 79.9	0.839
Serum creatinine, mg/dL (median [IQR])	4.6 (2.5 – 6.3)	3.3 (2.1 – 4.5)	0.277
Urea-creatinine ratio (median [IQR])	49.1 (40.8 – 95.1)	73.5 (44.8 – 112.5)	0.376
Serum sodium, mmol/L (mean ± SD)	132.7 ± 9.5	134.1 ±8.7	0.700
Serum potassium, mmol/L (median [IQR])	4.2 (4.0 – 5.0)	4.6 (4.00 – 5.35)	0.286
Ionized calcium, mmol/L (median [IQR])	1.16 (1.02 – 1.20)	1.04 (0.99 – 1.14)	0.042
Serum phosphorus, mg/dL (median [IQR])	5.10 (4.40 – 5.90)	5.90 (4.25 – 7.70)	0.521
Serum magnesium, mg/dL (median [IQR])	2.20 (2.00 – 3.10)	2.30 (2.05 – 2.60)	0.601
Serum bicarbonate, mmol/L (median [IQR])	20.7 (17.8 – 23.9)	20.9 (18.6 – 25.8)	0.517
Serum lactate, mmol/L (median [IQR])	2.1 (1.6 – 2.9)	2.40 (1.70 – 3.23)	0.421
D-dimer, mcg/dL (median [IQR])	2.34 (1.66 – 6.21)	3.37 (1.75 – 7.43)	0.334
C-reactive protein, mg/dL (median [IQR])	3.1 (1.9 – 4.5)	6.40 (3.95 – 18.60)	0.005
Troponin I, ng/mL (median [IQR])	0.06 (0 – 0.17)	0.15 (0.06 – 0.57)	0.043

## Discussion

In this sample of elderly, critically ill patients with COVID-19 who were treated with AKRT, in-hospital CFR was 93%, and most patients died within 10 days of diagnosis. This finding highlights how AKRT is a marker of severe illness and that healthcare professionals must be aware of the grave prognosis it implies.

The mortality in our study was higher than that of similar studies^25,28,30,31^. Gupta et al.^25^ conducted a multicenter cohort study of 3099 patients with critical COVID-19 that required ICU care within 67 hospitals across the United States of America, which found a CFR of 63% among AKRT patients. Zamoner et al.^30^ conducted a single-center, prospective, observational study in a public and tertiary university hospital in the city of São Paulo, consisting of 101 patients hospitalized with COVID-19. They found a CFR of 88% in AKRT patients. Samaan et al.^28^ conducted a multicenter, retrospective, observational study in 13 ICUs in the metropolitan region of the municipality of São Paulo, Brazil, consisting of 375 patients with AKRT associated with COVID-19. Their CFR was 72.5%, which can also be considered high, but lower than ours. The reasons for the difference between the studies are discussed in the following paragraphs.

The patients in our sample were predominantly elderly men with high prevalence rates of relevant comorbidities for COVID-19, especially systemic arterial hypertension (76%) and diabetes mellitus (44%). This shows that AKRT is used in COVID-19 patients with an already compromised health condition, including systemic diseases that may directly affect kidney function. Other studies had similar frequency of the aforementioned comorbidities, but their samples consisted of individuals with a median age younger than ours. Gupta et al.^25^, for instance, studied patients with a median age of 62 years (IQR 51 – 71); Zamoner et al.^30^ analyzed AKI patients with an average age of 61 ± 14.5 years. Samaan et al.^28^ analyzed individuals with a median age of 64 years (IQR 55 – 74), whereas in our study the median age was 74.5 years (IQR 64 – 82). Prevalence rates for hypertension and diabetes in study were similar to those in the studies conducted by Zamoner et al.^30^, Gupta et al.^25^, and Samaan et al.^28^, except for diabetes mellitus frequency in Zamoner et al.^30^, which was only 7% of AKI patients.

We did not notice any considerable differences between our patients and those in the studies conducted by Zamoner et al.^30^ and Samaan et al.^28^ regarding severity of illness assessed by the use of mechanical ventilation and vasopressors, but Gupta et al.^25^ had a sample that can be considered less critical than ours. Zamoner et al.^30^ reported that 76% of the AKI patients required IMV, similar to the percentage of individuals who were treated with vasoactive drugs. Samaan et al.^28^ found an 88.5% rate of IMV and an 85% of vasoactive drug use. The American research had a similar rate of IMV (79%), but a considerably lower rate of vasoactive drug use (51%). We have also found high mean or median levels of serum urea, creatinine, phosphorus, lactate, D-dimer, and C-reactive protein (CRP). The American study also showed higher levels of D-dimer and C-reactive protein, but lower values of serum creatinine. The latter also implies that our patients had worse renal function in comparison to this other research, as our patients were both older and had higher levels of serum creatinine.

As previously discussed, we found an overall hospital incidence of AKRT in COVID-19 patients of 6.7%. Considering the pandemic status of SARS-CoV-2 infection and the sheer number of COVID-19 cases, this may translate into a significant burden on nephrology services. These statistics might prove helpful for hospital administrators in planning and calculating the need for dialysis-related supplies. The only other study we were able to find that calculated the incidence of AKRT in all hospitalized patients found a frequency of 5.2%, a result we considered similar to ours^24^. In ICUs, the incidence varies from 15 to 20% of admitted patients, who frequently have predisposing conditions that lead to AKRT, like multiple organ dysfunction, shock, and cytokine release syndrome^25,28^. A systematic review with meta-analysis found an overall incidence of 9%, considering both the overall hospital admissions and ICU admissions^29^.

As far as we know, our study is the first to include a survival analysis of AKRT patients associated with COVID-19 in a hospital setting. We used the Kaplan-Meier estimator for graphical representation of survival over AKI duration in days. The median survival time of our patients was considerably short, which shows the critical state of our sample and the severity of AKI within the COVID-19 disease spectrum, specifically when treated with KRT. This reinforces the need for prevention of kidney dysfunction in moderate to severe cases of COVID-19 since it is a severe and life-threatening condition. Samaan et al.^28^ also found an AKI duration of 3 days for the patients who did not survive, which is identical to the findings of our study (3 days). Our group of non-expired individuals, however, revealed a median AKI duration of 23 days, while the group of surviving patients in the other study had a median AKI duration of 15 days. This difference can also be explained by the critical state of our sample.

As previously stated, the majority of patients expired. In univariate analysis, we found an association between survival and shorter ICU stay (p = 0.005), shorter AKI duration (p < 0.001), use of conventional hemodialysis (p = 0.028) and/or SLED (p = 0.002), KRT discharge (p < 0.001), levels of ionized calcium (p = 0.042), and lower levels of CRP (p = 0.005) and troponin I (p = 0.043). These results are biologically plausible and reasonable, as less time spent in ICU correlates with both lower frequency of hospital-related health problems and a lower severity of disease. Additionally, longer duration of AKI and absence of KRT discharge indicate severe kidney dysfunction and an overall more critical state of the disease. The use of intermittent hemodialysis (both conventional and SLED) also relates to survival because these patients tend to be more hemodynamically stable compared with those under CVVHDF. Finally, higher levels of CRP and troponin I suggest a more intense pro-inflammatory state and cardiac dysfunction, respectively.

We did not find any factors independently associated with death among COVID-19-associated AKRT. This could be explained by an insufficient sample size and considerable quantitative differences between expired and alive groups. Other studies have found association between death and age, severe oliguria, number of hospital beds, regional density of COVID-19 cases, KRT efficiency, and number of dysfunctional organs^25,31^.

Our research had several limitations. First, it was a retrospective review from a single center. Second, although we screened almost 1500 patients with COVID-19, our data is limited to the 100 patients who required AKRT; this relatively small sample size decreased the precision of our findings and hampered our evaluation of prognostic factors. Third, some of the variables studied were not uniformly registered in patients’ charts. Variables that were missing 5% or more of the information were excluded from the analysis. These variables were proteinuria, hematuria, and urinary casts (29% of missing data), 12-hour urine output (24%), and liver enzymes (14%). We also did not formally evaluate patients’ immune status and personal history of immunosuppression or transplantation but our sample had 44% of patients with diabetes, 12% with malignancy, and 4% with rheumatic diseases that probably had some degree of immunosuppression (due to underlying disease and/or its treatment), which could have contributed to the high mortality observed in this study. We also did not provide severity scores such as APACHE-II and SOFA; instead, we used need for vasoactive drugs and IMV as markers of disease severity. Finally, we did not have data on the indications for AKRT.

Nevertheless, this study provides important descriptive information regarding the incidence and CFR of AKRT in COVID-19 patients, a subject which has not been thoroughly researched. Moreover, as far as we know, this is the first study to evaluate this clinical condition in the state of Bahia, Brazil. Other similar studies have been conducted in Brazilian territory, but in a different population, specifically from the state of São Paulo. The publication of similar studies from different parts of our country would result in a better understanding of the actual incidence and mortality of AKRT in COVID-19 throughout Brazil.

## Conclusion

AKRT was used in 6.7% of patients hospitalized with COVID-19, 93% of which expired. AKRT patients were typically older, critically ill, and most died within 10 days of diagnosis. This finding shows that AKI, especially when KRT is needed, is a serious complication of SARS-CoV-2 infection and should not be overlooked by health services and that better strategies to address this issue are urgently needed.
